# Evaluating Factors Affecting Mean Glandular Dose in Mammography: Insights from a Retrospective Study in Dubai

**DOI:** 10.3390/diagnostics14222568

**Published:** 2024-11-15

**Authors:** Kaltham Abdulwahid Mohammad Noor, Norhashimah Mohd Norsuddin, Muhammad Khalis Abdul Karim, Iza Nurzawani Che Isa, Vaidehi Ulaganathan

**Affiliations:** 1 Diagnostic Imaging & Radiotherapy Program, Faculty of Health Sciences, The National University of Malaysia (UKM), Kuala Lumpur 50300, Malaysia or kanoor@dubaihealth.ae (K.A.M.N.); zawaniisa@ukm.edu.my (I.N.C.I.); 2 Dubai Health Academic Corporation (DHAC), Rashid Hospital, Radiology Department, Dubai, United Arab Emirates; 3 Department of Physics, Faculty of Science, University Putra Malaysia (UPM), Serdang 43400, Malaysia; mkhlis@upm.edu.my; 4 Faculty of Applied Science, Uplands College of Science and Technology Incorporated (UCSI), Kuala Lumpur 56000, Malaysia; vaidehi@ucsiuniversity.edu.my

**Keywords:** mean glandular dose, mammography screening, exposure parameters, compression force, breast density

## Abstract

Background/Objective: This study evaluates the mean glandular dose (MGD) in mammography screening for women aged 40–69 in Dubai, based on a retrospective analysis of a dose survey involving 2599 participants. Methods: MGD was calculated using the Dance formula. Results: The average MGD was 0.96 ± 0.39 mGy for mediolateral oblique (MLO) views and 0.81 ± 0.33 mGy for craniocaudal (CC) views. Weak inverse correlations were found between age and organ dose (OD) for both views, while a direct relationship was observed between breast thickness and entrance skin dose (ESD). In adjusted models, ESD was strongly associated with MGD (β = 1.04, 95% CI: 0.97, 1.09), while OD showed a moderate association (β = 0.44, 95% CI: 0.40, 0.49). Significant variations in ESD, OD, and MGD were noted across age groups and breast thicknesses. Conclusions: Lower MGD indicates reduced radiation exposure risk, while higher MGD in MLO views suggests improved imaging quality. Monitoring and optimizing MGD are essential for enhancing patient safety and screening efficacy.

## 1. Introduction

Breast cancer is a major global public health issue, and mammography plays a crucial role in early identification, leading to a decrease in mortality rates [1]. Nonetheless, screening should be weighed against the potential risks, particularly the radiation dose absorbed by breast tissue during imaging [2]. In line with this, the mean glandular dose (MGD) is an important dosimetry parameter needed to approximate the dose acquired by the breast tissues during mammographic imaging [3]. A critical dosimetry parameter that calculates the radiation dose absorbed by breast tissue during mammographic imaging is the mean glandular dose (MGD). It is fundamental to understand MGD to establish secure and effective screening protocols, as the risk of radiation-induced cancers can be elevated by excessive radiation exposure. Therein lies the importance of studying the factors that influence MGD to enhance mammography practices [4].

Dubai is an Emirate of the United Arab Emirates that is growing quickly and has experienced significant population growth and progresses in healthcare services over the past few decades, including the implementation of breast cancer screening programs. At the same time, despite these developments, recent research on MGD levels and influencing factors within Dubai’s diverse population remains limited. By assessing MGD levels in women between the ages of 40 and 69 and pinpointing important factors influencing these radiation doses, this study seeks to close this gap. This study aimed to bridge the gap regarding the MGD levels in the female population in Dubai and the affecting variables. One of the significant elements affecting MGD is breast density; dense breasts absorb more radiation and thus require more dose to acquire clear images. However, other variables also play a critical role in determining MGD. Breast thickness, compressive force, image acquisition strategies, and parameters associated with X-ray machines comprise this category. It is vital to optimize these variables to obtain clear and valuable images while minimizing radiation exposure. The objective of this investigation is to assess the influence of these critical variables on MGD among the female population of Dubai. In this study, we offer valuable insights into the determinants of MGD levels in this demographic by analyzing the influence of breast thickness, compressive force, and X-ray machine parameters. It is essential to understand the relationship between these variables to create mammography protocols that are safe, effective, and customized to the distinctive characteristics of the Dubai population [5]. Improving these variables will help to acquire clear and useful images and minimize radiation dose [6]. This study utilized a retrospective analysis of mammographic images and attended patient data from multiple breast screening centers in Dubai. The patient cohort was selected to be representative and included females of different ages and geographical regions. Subsequently, MGD dosimetry measurements were calculated using the Dance formula [7,8], and the results were subjected to statistical analysis to identify the possible correlation with age, breast thickness, compression force, and other affecting variables.

This study seeks to provide vital advice to radiologists and policymakers by evaluating the MGD levels in the female population in Dubai. The findings will aid in formulating effective screening techniques tailored to the unique demographic characteristics of Dubai. The insights gained from this study have the potential to contribute to the current efforts to improve the efficiency of breast cancer screening while concurrently reducing the hazards associated with radiation exposure. Eventually, the findings of this study will definitely influence clinical practice by shaping policy guidelines and ultimately contribute to updated evidence-based patient care in regard to mammography screening practice in Dubai and possibly even further abroad.

## 2. Materials and Methods

This cross-sectional retrospective study received ethical approval from both the Dubai Scientific Research Ethics Committee (DSREC-SR-08/2022_04) and the Medical Research and Ethics Committee at the National University of Malaysia UKM (JEP-2022-622).

### 2.1. Study Design and Data Collection

The dose survey data of patients who had mammographic procedures from November 2019 to November 2022 were collected retrospectively. A total of 2599 mammograms of women aged 40–69 years were exported from the system.

### 2.2. Instruments and Software

This study utilized five Siemens mammography machines (Mammo inspiration). Dose survey data were exported via PACS team in Radiology Department in one of the government hospitals in Dubai using the DOSE TQM system (Qaelum NV, Leuven, Belgium) and extracted into SPSS software version 25.0 for inferential analysis.

### 2.3. Inclusion and Exclusion Criteria

Since this study is targeting the breast screening program, the inclusion criteria were female patients aged 40–69 years who underwent routine mammography screening during the study period. Exclusion criteria included patients with incomplete mammography, repeated exams, extra views like magnified view and mediolateral view, and patients with breast implants. The reasons for excluding these cases were that they can have a significant effect on the mean glandular dose (MGD) measurements by increasing or decreasing the actual expected value of MGD [9,10]. Therefore, this study focuses on standard patients to ensure that the results reflect average MGD levels without being confounded by these variables [11].

### 2.4. Data Extraction and Variables

Patient demographics, mammographic images, and relevant technical parameters were extracted from the patients’ imaging archives of the selected screening clinics. The following information was compiled for each mammogram: patient’s age, breast thickness, compression force, X-ray machine parameters, and imaging technique.

**Figure 1:**
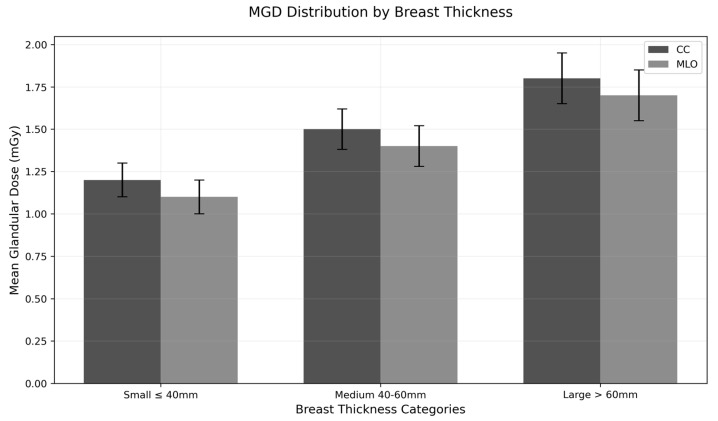
Mean glandular dose distributions by breast thickness for CC and MLO views.

Mean glandular dose (*MGD*) values were calculated using the following formula [12]:

*MGD* = *K* × *g* × *c* × *s*

where *MGD* represents the mean glandular dose; *K* is the incident air kerma; *g* is the granularity coefficient accounting for the proportion of glandular tissue within the breast; *c* is the calibration coefficient specific to the utilized mammography system; and *s* is the backscatter factor reflecting the backscatter radiation directed back to the breast [13]. Then, the concluded *MGD*s were categorized based on three different breast thicknesses, as shown in Figure 1.

### 2.5. Statistical Analysis

The Statistical Packages for the Social Sciences (SPSS) software version 28.0 (Armonk, NY, USA was used to conduct the statistical analysis. Means and standard deviations were computed for each variable. Correlation analysis was implemented to study correlations between mean glandular dose (MGD) and variables counting age, breast thickness, and compressive force, while analysis of variance (ANOVA) was implemented to evaluate variability in MGD levels across various breast thickness categories. The relationships between input parameters (age, breast thickness, acquisition parameters) and output parameters (ESD, OD, and MGD) were evaluated using linear regression analysis. Additionally, polynomial regression was applied to model the relationship between mAs and MGD due to the non-linear nature of this relationship. Statistical significance was set at *p* < 0.05 for all analyses.

## 3. Results

### 3.1. Demographics and Clinical Characteristics

The mean age of the participants was 53.4 ± 8.6 years. The distribution of participants by age group was as follows: 480 (18.5%) were under 50 years, 1560 (60.0%) were between 50 and 60 years, and 559 (21.5%) were over 60 years. The mean breast thickness was 48.3 ± 16.4 mm, with 510 (19.6%) having small breasts (≤30 mm), 1560 (60.0%) medium breasts (40–60 mm), and 529 (20.4%) large breasts (≥70 mm).

Table 1 summarizes the distribution of ESD, OD, and MGD across breast thickness and age groups for both craniocaudal (CC) and mediolateral oblique (MLO) views. The overall mean ESD was higher for the MLO view (5.68 ± 2.57 mGy) compared to the CC view (4.66 ± 1.94 mGy). ESD significantly increased with breast thickness for both CC (F = 218.9, *p* < 0.001) and MLO (F = 573.7, *p* < 0.001) views. However, ESD significantly decreased with age for both CC (F = 44.39, *p* < 0.001) and MLO (F = 46.39, *p* < 0.001) views. The overall mean OD was also higher for the MLO view (1.38 ± 0.45 mGy) compared to the CC view (1.22 ± 0.38 mGy). OD significantly increased with breast thickness for both CC (F = 48.3, *p* < 0.001) and MLO (F = 176.0, *p* < 0.001) views. However, OD significantly decreased with age for both CC (F = 67.70, *p* < 0.001) and MLO (F = 63.91, *p* < 0.001) views. The overall mean MGD was higher for the MLO view (0.96 ± 0.39 mGy) compared to the CC view (0.81 ± 0.33 mGy). MGD showed insignificant increases with breast thickness for the CC view (F = 2.70, *p* = 0.067), but there was a significant increase for the MLO view (F = 53.5, *p* < 0.001). MGD significantly decreased with age for both CC (F = 28.99, *p* < 0.001) and MLO (F = 34.65, *p* < 0.001) views. Figure 1 displays the estimated MGD for three different breast thickness categories.

**Figure 2:**
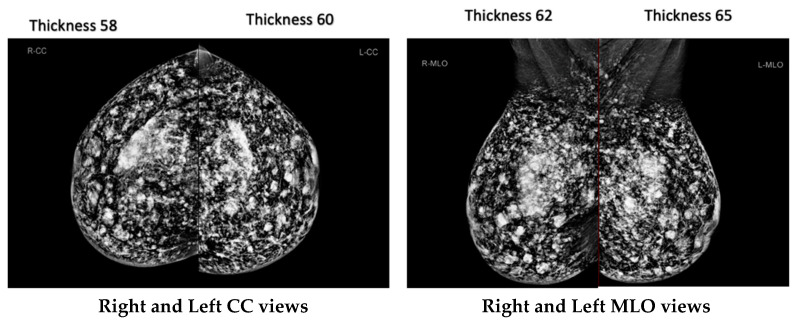
Mammography images for the same patient, showing different breast thicknesses (58 mm, 60 mm, 62 mm, 65 mm).

Figure 2 shows mammograms of the same patient with varying breast thicknesses (58 mm, 60 mm, 62 mm, and 65 mm) due to different mammographic views (CC and MLO). As breast thickness increases, there is a corresponding increase in MGD. For smaller breasts (<40 mm), MGD values remain comparatively steady between craniocaudal (CC) and mediolateral oblique (MLO) projections. However, as breast thickness increases into the medium range (40–60 mm), MGD values rise more significantly, particularly in MLO views compared to CC views. The highest MGD measurements are detected in large breasts (>60 mm), where MLO views consistently show higher doses than CC projections. This suggests a direct correlation between tissue volume and radiation absorption patterns, with thicker breasts requiring higher doses for clear imaging.

### 3.2. Regression Analysis

Table 2 shows the association of age, breast thickness, and acquisition parameters with ESD, OD, and MGD. Age showed an inverse association with ESD, OD, and MGD in both MLO and CC views. The regression analysis indicated the highest association between age and OD for both MLO (β = −0.22, 95% CI: −0.25, −0.18) and CC (β = −0.24, 95% CI: −0.27, −0.20). Positive moderate associations were observed between breast thickness and the average ESD (MLO: β = 0.65, 95% CI: 0.62, 0.67; CC: β = 0.60, 95% CI: 0.57, 0.63) and OD (MLO: β = 0.40, 95% CI: 0.37, 0.43; CC: β = 0.31, 95% CI: 0.28, 0.35). A weak association was identified between breast thickness and MGD (MLO: β = 0.24, 95% CI: 0.20, 0.28; CC: β = 0.19, 95% CI: 0.15, 0.22). The X-ray tube voltage (kVp) showed a significant association with ESD for both MLO (β = 0.57, 95% CI: 0.54, 0.60) and CC (β = 0.54, 95% CI: 0.50, 0.57) views. The exposure (mAs) was significantly associated with OD for both MLO (β = 0.86, 95% CI: 0.84, 0.88) and CC (β = 0.78, 95% CI: 0.76, 0.81) views.

### 3.3. Correlation Analysis

**Figure 3:**
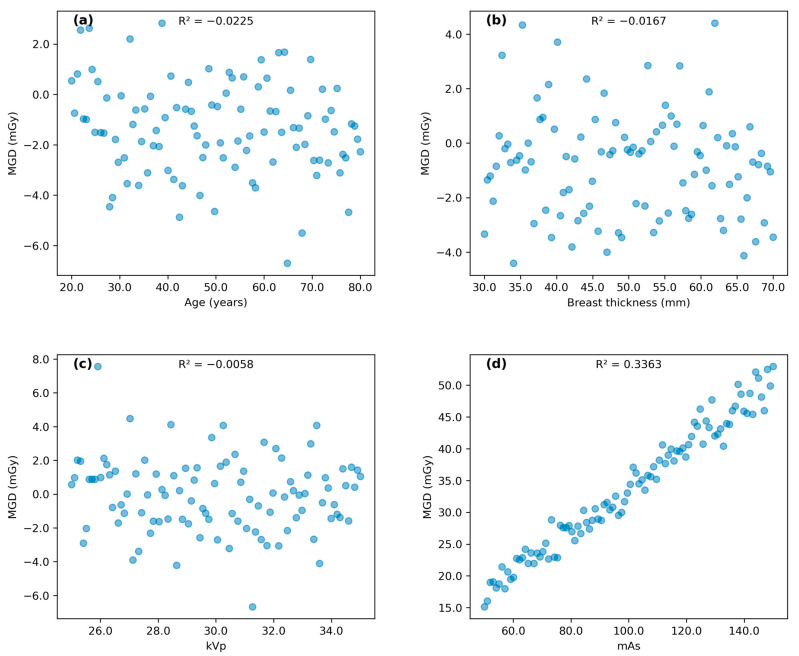
Scatter plots illustrating correlation pattern between (**a**) subject’s age, (**b**) breast thickness, (**c**) kVp, and (**d**) mAs with MGD for MLO view.

**Figure 4:**
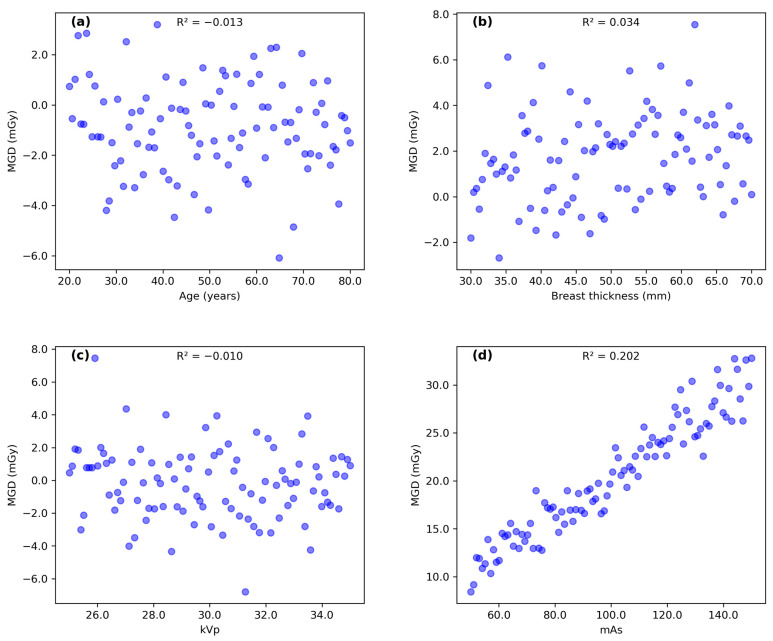
Scatter plots illustrating correlation pattern between (**a**) subject’s age, (**b**) breast thickness, (**c**) kVp, and (**d**) mAs with MGD for CC view.

Figure 3 and Figure 4 illustrate the correlation patterns between age, breast thickness, kVp, and mAs with MGD for MLO and CC views, respectively. MGD in MLO view showed an indirect correlation with age (R^2^ = 0.025) and direct correlations with breast thickness (R^2^ = 0.057), kVp (R^2^ = 0.008), and mAs (R^2^ = 0.282). The coefficient of determination (R^2^) values in this study represents the proportion of variance in mean glandular dose (MGD) that can be explained by each variable. R^2^ values range from 0 to 1, with values closer to 1 indicating stronger correlations. A similar pattern was observed for the CC view, with MGD showing an indirect correlation with age (R^2^ = 0.021) and direct correlations with breast thickness (R^2^ = 0.034), kVp (R^2^ = 0.010), and mAs (R^2^ = 0.209).

### 3.4. Linear Regression Analysis

Table 3 presents the linear regression analysis between ESD and OD as input parameters and MGD for each view. In the unadjusted model, both ESD and OD showed a strong correlation with MGD. After adjusting for confounding variables, ESD remained strongly associated with MGD (MLO: β = 1.04, 95% CI: 0.97, 1.09; CC: β = 1.02, 95% CI: 0.96, 1.08), while OD was moderately associated with MGD (MLO: β = 0.44, 95% CI: 0.40, 0.49; CC: β = 0.30, 95% CI: 0.24, 0.35).

### 3.5. Polynomial Regression Analysis

**Figure 5:**
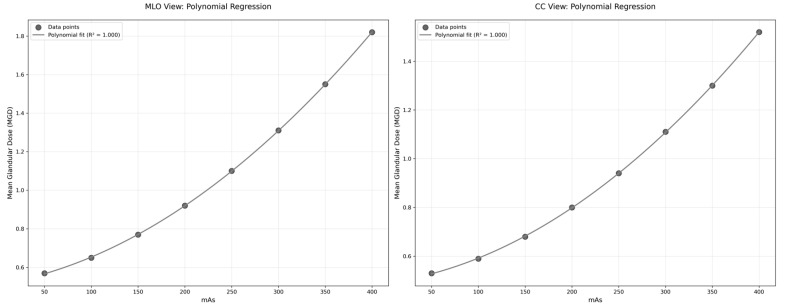
Polynomial regression analysis of the relationship between mAs and mean glandular dose (MGD) for MLO and CC views.

We moved beyond conventional linear analysis and explored polynomial regression analysis to verify the connection between mAs and MGD, which was particularly noteworthy. Traditional linear modeling showed limited correlation, but polynomial regression proved stronger underlying patterns. In MLO projections, the coefficient of determination strengthened considerably from 0.282 to 0.807 when applying a second-order fit [MGD = 0.00001(mAs)^2^ + 0.00569(mAs) + 0.535077]. CC view analysis showed similar improvement, with R^2^ increasing from 0.209 to 0.686 [MGD = 0.00001(mAs)^2^ + 0.006074(mAs) + 0.488528]. These findings, illustrated in Figure 5, demonstrate that radiation dose increases more rapidly at higher mAs settings, following a curved trace rather than a simple proportional relationship. The quadratic fit demonstrates a stronger correlation compared to linear regression, with R^2^ values of 0.807 for MLO and 0.686 for CC, indicating a non-linear relationship between mAs and MGD. This non-linear pattern better symbolizes the complex relationship between exposure factors and tissue dose in mammographic imaging.

## 4. Discussion

The decreased mean glandular dose (MGD) values that were revealed in this study have essential implications for the practice of mammography in Dubai and possibly even further afield. It is possible to reconsider screening techniques because the average MGD values for MLO are 0.96 ± 0.39 mGy, while the average MGD values for CC views are 0.81 ± 0.33 mGy. These values are significantly lower than the European standard level of 2.5 mGy. Because of these lower doses, it may be possible to justify starting the first screening mammography investigation one or two years earlier than forty, as the number of breast cancer cases is in a steady increase globally.

While some correlations were weak (R^2^ < 0.1), such as those between age and MGD (R^2^ = 0.025 for MLO and R^2^ = 0.021 for CC), they still provide valuable insights into the complex relationships between various factors and MGD. The weak correlations may be due to the multifactorial nature of MGD determination or the presence of confounding variables not accounted for in this analysis. The strongest correlation was observed between mAs and MGD (R^2^ = 0.282 for MLO and R^2^ = 0.209 for CC), suggesting that mAs is a significant factor influencing radiation dose in mammography. The polynomial regression analysis revealed a more complex relationship between mAs and MGD than initially indicated by linear regression. The substantial improvement in R^2^ values (0.807 for MLO and 0.686 for CC views) suggests that the relationship between these parameters follows a quadratic pattern rather than a simple linear correlation. This non-linear relationship aligns with theoretical expectations of radiation physics, where the interaction between X-ray production and tissue absorption characteristics typically follows complex patterns. The similar quadratic coefficients (0.00001) observed in both MLO and CC views indicate consistency in the underlying physical relationship, while the different linear terms likely reflect variations in tissue thickness and compression between views. These findings provide valuable insights for dose optimization protocols, suggesting that linear adjustments of mAs may not result in proportional changes in MGD, particularly at higher mAs values where the quadratic effect becomes more noticeable.

Breast thickness can vary significantly between different mammographic views, even within a single patient (Figure 2). This variability highlights the importance of tailoring imaging protocols to account for individual anatomical differences. Customizing these protocols ensures that radiation dose is optimized while maintaining image quality. It is crucial to recognize that the immediate risk associated with a single screening may decrease as one ages; however, cumulative radiation exposure from multiple screenings can still result in substantial health risks over time. Accumulated doses could increase the likelihood of radiation overexposure. The relationship between increased screening frequency and improved detection rates is not always linear. There is a higher potential for overdiagnosis and overtreatment of slow-growing or clinically insignificant cancers among older women, which may lead to unnecessary interventions without a proportional survival benefit, ultimately impacting their overall health and quality of life. As an additional point of interest, the fact that MGD varies across different breast thicknesses underscores the requirement of developing personalized imaging protocols. It is possible to further optimize dosage levels while retaining diagnostic accuracy by implementing automated exposure control systems that modify settings based on the features of each individual breast.

This work makes a considerable contribution to the understanding of the methodology for determining radiation dosage in mammography and the aspects related to it. The findings suggest that the mean glandular dose (MGD) is a dependable metric for assessing the radiation dosage [4,14], exhibiting lower values in comparison to the entrance skin dose (ESD) and organ dose (OD), hence reducing the likelihood of radiation exposure. The results align with prior research conducted by Salomon et al. [15] and Al-Naemi et al. [16], which also observed differences in MGD depending on breast thickness and age. The findings of the present investigation are consistent with the results of the Digital Mammographic Imaging Screening Trial (DMIST) carried out by the American College of Radiology Imaging Network (ACRIN), which indicated an average mean glandular dose (MGD) of 3.7 mGy (1.86 mGy per view) [17,18]. The lower average MGD observed in this study for both MLO and CC views (0.96 ± 0.39 mGy vs. 0.81 ± 0.33 mGy) is consistent with findings from other research in this area [13,14,15,19,20,21]. Breast compression and other factors such as kVp and mAs were found to significantly influence MGD [22,23]. This study observed a positive correlation between breast thickness and MGD, consistent with previous findings by Alizadeh Riabi et al. [24] and Du et al. [25]. Age was an additional noteworthy variable, as elderly participants exhibited a reduced MGD, which aligns with the results reported by Baek et al. [26] and Pwamang et al. [27].

Future research in this field should prioritize several key areas. Initially, longitudinal studies monitoring MGD levels over time may uncover significant trends linked to technical breakthroughs or modifications in screening techniques [28]. Such studies could provide valuable insights into the long-term effects of dose optimization strategies and help predict future dose levels. Secondly, the application of artificial intelligence (AI) in mammography dose management presents an exciting opportunity for research. Exploring AI’s potential in optimizing exposure parameters and predicting MGD could lead to the development of real-time dose management systems [29,30]. These advanced systems could aid in adjusting imaging parameters based on individual patient characteristics, potentially further improving both patient safety and image quality. Ultimately, investigating the use of newer imaging modalities, such as contrast-enhanced spectral mammography or digital breast tomosynthesis, under optimized dosage regulations may provide enhancements in breast cancer detection while ensuring minimal radiation exposure. Such findings could facilitate the development of comprehensive, low-dose breast imaging methods that optimize diagnostic efficacy while reducing patient risk.

## 5. Limitation

This study has several limitations that should be acknowledged. To start with, the focus of this investigation was on mammography dose-related parameters but not the density of breast tissues. Although we examined several variables, including age, breast thickness, compression force and dose parameters, this study was not designed to address other technical issues that can affect Mean Glandular Dose (MGD). These include variances in radiographer technical experience, breast density as well as differences in mammography vendors. Secondly, we assumed that all mammography images were of sufficient diagnostic quality without explicitly evaluating image quality metrics. This assumption may have overlooked potential variations in image quality that could impact dose requirements. Furthermore, our retrospective design, while allowing for a large sample size, excluded the collection of certain patient medical data that might influence radiation dose, such as body mass index or history of previous breast surgeries. To address these limitations, future research should incorporate breast density assessments, possibly using automated volumetric density measurement tools. Including details of clinical history of the disease for each patient might also help researchers to draw a clear picture about the status of how mammography investigation can help the journey of breast cancer diagnosis. Moreover, our research was restricted to a single vendor’s equipment, which might not accurately reflect the variety of mammography systems available. A multi-vendor study might offer more comprehensive understanding of the ways in which various technologies can impact radiation exposure.

## 6. Conclusions

This study shines a spotlight on the mean glandular dose (MGD) and its variations during mammography screenings for women aged 40 to 69 in Dubai. Our findings reveal that radiation exposure levels, including entrance skin dose (ESD) and MGD, are influenced by breast thickness and age, with MLO views consistently showing higher doses than CC views. Notably, the observed MGD values remain well below international safety standards, suggesting that current screening protocols effectively minimize radiation risks while maintaining diagnostic accuracy. To further enhance patient safety and optimize breast cancer screening programs, we recommend the regular monitoring and adjustment of MGD levels. Tailoring mammography protocols to account for individual factors such as age and breast thickness will help reduce radiation exposure without compromising image quality. These findings have the potential to inform national health guidelines and improve mammography practices not only in Dubai but also globally.

Future research should focus on multivariate analyses to explore the relationship between factors affecting MGD more comprehensively. By refining dose management strategies and incorporating advanced technologies, healthcare professionals can improve early detection rates while minimizing radiation-related risks, ultimately contributing to better patient outcomes worldwide.

## Figures and Tables

**Table 1:** Descriptive summary of entrance skin dose (ESD), organ dose (OD), and mean glandular dose (MGD) based on view, breast thickness, and age group.

View	CC			MLO		
	Mean ± SD	(Min-Max)	F (*p*)	Mean ± SD	(Min–Max)	F (*p*)
	Entrance Dose (mGy)					
Overall	4.66 ± 1.94	(0–22.36)		5.68 ± 2.57	(0–24)	
Breast thickness						
Small (≤30 mm)	2.56 ± 1.18	(0–7.22)	218.9 (<0.001)	2.52 ± 1.01	(0–6)	573.7 (<0.001)
Medium (40–60 mm)	4.36 ± 1.67	(0–13.71)		5.00 ± 1.88	(0–16)	
Large (≥70 mm)	5.74 ± 2.17	(0–22.36)		7.89 ± 2.84	(0–24)	
Age (years)						
<50	4.99 ± 2.10	(0–22.36)	44.39 (<0.001)	6.04 ± 2.74	(0–24)	46.39 (<0.001)
50–60	4.57 ± 1.80	(0–13.71)		5.68 ± 2.44	(0–23)	
>60	4.01 ± 1.64	(0–9.58)		4.69 ± 2.12	(0–16)	
	Organ Dose (mGy)					
Overall	1.22 ± 0.38	(0.55–3.64)		1.38 ± 0.45	(0.57–4.43)	
Breast thickness						
Small (≤30 mm)	1.01 ± 0.28	(0.57–1.69)	48.3 (<0.001)	0.99 ± 0.28	(0.57–2.26)	176.0 (<0.001)
Medium (40–60 mm)	1.19 ± 0.36	(0.55–3.51)		1.31 ± 0.41	(0.58–3.55)	
Large (≥70 mm)	1.32 ± 0.40	(0.70–3.64)		1.62 ± 0.49	(0.82–4.43)	
Age (years)						
<50	1.30 ± 0.40	(0.66–3.64)	67.70 (<0.001)	1.47 ± 0.47	(0.66–4.01)	63.91 (<0.001)
50–60	1.19 ± 0.36	(0.55–3.51)		1.36 ± 0.44	(0.57–4.43)	
>60	1.07 ± 0.29	(0.55–2.12)		1.19 ± 0.34	(0.61–3.27)	
	Mean Glandular Dose (mGy)					
Overall	0.81 ± 0.33	(0–2.47)		0.96 ± 0.39	(105–5.14)	
Breast thickness						
Small (≤30 mm)	0.86 ± 0.36	(0–1.98)	2.70 (0.067)	0.85 ± 0.35	(104–2.82)	53.5 (<0.001)
Medium (40–60 mm)	0.81 ± 0.33	(0–2.61)		0.92 ± 0.34	(41.0 × 104)	
Large (≥70 mm)	0.79 ± 0.33	(0–2.47)		1.09 ± 0.47	(2.0 × 104)	
Age (years)						
<50	0.86 ± 0.33	(0–2.47)	28.99 (<0.001)	1.02 ± 0.40	(1.2 × 104)	34.65 (<0.001)
50–60	0.77 ± 0.30	(0–2.61)		0.95 ± 0.36	(1.3 × 104)	
>60	0.75 ± 0.37	(0–2.52)		0.96 ± 0.39	(5.9 × 105)	

**Table 2:** Evaluation of regression analyses between subject’s age, breast thickness, and acquisition parameters as input parameters and entrance dose (ESD), organ dose (OD), and mean glandular dose (MGD) as output parameters for each view.

Variable	Output Parameter: Entrance Dose (mGy)					
	MLO			CC		
	β	95% CI	*p*-Value	β	95% CI	*p*-Value
Age (years old)	−0.18	−0.22, −0.15	<0.001 *	−0.20	−0.23, −0.16	<0.001 *
Breast thickness (mm)	0.65	0.62, 0.67	<0.001 *	0.60	0.57, 0.63	<0.001 *
X-ray tube voltage (kVp)	0.57	0.54, 0.60	<0.001 *	0.54	0.50, 0.57	<0.001 *
Exposure (mAs)	0.85	0.83, 0.87	<0.001 *	0.79	0.77, 0.81	<0.001 *
All input parameters (listed above)	−0.09	−0.13, −0.06	<0.001 *	−0.16	−0.19, −0.12	<0.001 *
	**Output Parameter: Organ Dose (mGy)**					
	**MLO**			**CC**		
	β	95% CI	*p*-value	β	95% CI	*p*-value
Age (years old)	−0.22	−0.25, −0.18	<0.001 *	−0.24	−0.27, −0.20	<0.001 *
Breast thickness (mm)	0.40	0.37, 0.43	<0.001 *	0.31	0.28, 0.35	<0.001 *
X-ray tube voltage (kVp)	0.33	0.29, 0.36	<0.001 *	0.26	0.22, 0.29	<0.001 *
Exposure (mAs)	0.86	0.84, 0.88	<0.001 *	0.78	0.76, 0.81	<0.001 *
All input parameters (listed above)	−0.07	−0.10, −0.03	0.001 *	−0.12	−0.16, −0.08	<0.001 *
	**Output Parameter: Mean Glandular Dose (mGy)**					
	**MLO**			**CC**		
	β	95% CI	*p*-value	β	95% CI	*p*-value
Age (years old)	−0.16	−0.20, −0.12	<0.001 *	−0.14	−0.18, −0.11	<0.001 *
Breast thickness (mm)	0.24	0.20, 0.28	<0.001 *	0.19	0.15, 0.22	<0.001 *
X-ray tube voltage (kVp)	0.09	0.05, 0.13	<0.001 *	0.10	0.07, 0.14	<0.001 *
Exposure (mAs)	0.53	0.50, 0.56	<0.001 *	0.46	0.42, 0.49	<0.001 *
All input parameters (listed above)	−0.04	−0.08, −0.01	0.028 *	−0.07	−0.11, −0.03	<0.001 *

MLO: Mediolateral oblique view, CC: Craniocaudal view; *: *p*-values marked with an asterisk (*) indicate statistically significant results at the 0.05 level (*p* < 0.05), suggesting a strong likelihood that the observed effects are not due to chance.

**Table 3:** Linear regression analysis between entrance skin dose (ESD) and organ dose (OD) as input parameters and mean glandular dose (MGD) for each view.

Variable	Output Parameter: Mean Glandular Dose (mGy)					
	MLO			CC		
	β	95% CI	*p*-Value	β	95% CI	*p*-Value
Entrance Dose (mGy)	0.78	0.75, 0.80	<0.001 *	0.78	0.75, 0.80	<0.001 *
Organ Dose (mGy)	0.80	0.78, 0.82	<0.001 *	0.79	0.77, 0.82	<0.001 *
Entrance Dose (mGy)	1.04	0.97, 1.09	<0.001 *	1.02	0.96, 1.08	<0.001 *
Organ Dose (mGy)	0.44	0.40, 0.49	<0.001 *	0.30	0.24, 0.35	<0.001 *

MLO: Mediolateral oblique view, CC: Craniocaudal view. *: *p*-values marked with an asterisk (*) indicate statistically significant results at the 0.05 level (*p* < 0.05), suggesting a strong likelihood that the observed effects are not due to chance.

## Data Availability

The data presented in this study are available on request from the corresponding author. The data are not publicly available for ethical purposes.

## Abbreviations

CC	Craniocaudal view
MLO	Mediolateral view
ESD	Entrance skin dose
kVp	Kilovolt peak
mAs	Milliampere-seconds
MGD	Mean glandular dose
OD	Organ dose
PACS	Picture archiving computerized system
